# Alternative splicing of mRNA in colorectal cancer: new strategies for tumor diagnosis and treatment

**DOI:** 10.1038/s41419-021-04031-w

**Published:** 2021-07-30

**Authors:** Yanyan Chen, Mengxi Huang, Xiaolong Liu, Yadi Huang, Chao Liu, Jialong Zhu, Gongbo Fu, Zengjie Lei, Xiaoyuan Chu

**Affiliations:** 1 Department of Medical Oncology, Jinling Hospital, Medical School of Nanjing University, Nanjing University, Nanjing, China; 2 Department of Cardiothoracic Surgery, Jinling Hospital, Nanjing Medical University, Nanjing, China; 3 Department of Medical Oncology, Jinling Hospital, First School of Clinical Medicine, Southern Medical University, Nanjing, China; 4 Department of Medical Oncology, Jinling Hospital, Nanjing Medical University, Nanjing, China

**Keywords:** Cancer epidemiology, Cell biology, Molecular biology

## Abstract

Alternative splicing (AS) is an important event that contributes to posttranscriptional gene regulation. This process leads to several mature transcript variants with diverse physiological functions. Indeed, disruption of various aspects of this multistep process, such as cis- or trans- factor alteration, promotes the progression of colorectal cancer. Therefore, targeting some specific processes of AS may be an effective therapeutic strategy for treating cancer. Here, we provide an overview of the AS events related to colorectal cancer based on research done in the past 5 years. We focus on the mechanisms and functions of variant products of AS that are relevant to malignant hallmarks, with an emphasis on variants with clinical significance. In addition, novel strategies for exploiting the therapeutic value of AS events are discussed.

## Facts

1. Pre-mRNA alternative splicing events increase the complexity of gene regulation and transcript diversity
2. Variable cis-regulatory elements and alterations in splicing trans-regulatory factors regulate alternative splicing events
3. Aberrant alternative splicing influences colorectal cancer progression by regulating the cell proliferation, invasion, and apoptosis, as well as angiogenesis and drug-resistance
4. Antisense oligonucleotides and small molecule inhibitors are therapeutic agents based on splicing alterations
5. Further studies are needed to enhance the targeting efficiency of antisense oligonucleotides in CRC patients.

## Open questions

1. Alternative splicing is an important molecular event contributing to post-transcription regulation. What kinds of abnormal changes in splicing sequence and regulatory factors lead to splicing variant alterations in colorectal cancer?
2. Alterations of alternative splicing result in several products that are carcinogenic. What role do the products play in colorectal cancer?
3. Alternative splicing provides new targets for cancer treatment. Does targeting the multi-steps of alternative splicing provide important clinical value?

## Introduction

Alternative splicing (AS) of precursor messenger RNA (pre-mRNA) has been shown to influence physiological and pathological processes. Pre-mRNA splicing was first discovered in 1977, and has now been shown to play important roles in posttranscriptional regulation of gene expression [1]. Notably, AS increases the diversity of transcript variants and proteomic isoforms. A recent analysis of the Encyclopedia of DNA Elements (ENCODE) project 1 (GRCh38, Ensembl79) and data from recent research have revealed that the human genome consists of ~21,306 protein-coding genes [2, 3], but the number of transcript variants and protein isoforms is considerably higher because of AS [4]. Alternative and aberrant pre-mRNA splicing have the potential to act as diagnostic and treatment targets, especially in primary and metastatic tumors [5].

Colorectal cancer (CRC) has been reported to have the third-highest mortality and morbidity rates in the latest epidemic oncology study in the United States. Investigations into the fundamental pathological mechanisms of CRC have revealed that AS events can be exploited to offer more diagnostic and treatment agents for cancer [6]. The first study on AS in CRC revealed that c-Ki-ras (KRAS), a protein-coding gene, mutates at splice acceptor site and produces two transcript variants with exon 4A included or excluded in SW480 colon carcinoma cell lines [7]. Accumulating evidence has confirmed that some agents targeting AS events can effectively treat CRC by restoring aberrant alternative splicing procedures, such as splice-switching oligonucleotides (SSOs) targeting specific sequences and inhibitors like indacaterol that targets splicing regulatory proteins in cancer.

Several review articles have shown that AS is related to epigenetic events [8], therapeutic targets [9], and splicing variants of particular classical molecules, such as B-Raf proto-oncogene (BRAF) [10] and vascular endothelial growth factor A (VEGF-A) in CRC [11]. In this review, we highlight recent advances (in the past 5 years) in the identification and characterization of AS events in CRC. We also describe the roles of mRNA splicing and aberrant regulation of AS in CRC. In addition, this review consolidates the biological functions of AS and splicing products, as well as the current efforts to develop its potential of clinical application in the diagnosis or treatment of cancer.

## Splicing machinery and alternative splicing

A sophisticated spliceosome machinery is responsible for splicing pre-mRNA by recognizing different splice sites. This machinery catalyzes two fundamental transesterification reactions in the nucleus [12]. The process of pre-mRNA splicing is regulated by specific recognition between cis-regulatory elements (pre-mRNA sequence) and trans-regulatory factors (proteins in splicing process). This is followed by cutting and joining of ribonucleotide base sequences [13]. In the review, we discuss the mechanisms by which the spliceosome routinely removes introns and joins exons to generate a mature mRNA and the classical types of AS.

### The spliceosome machinery

The core spliceosome comprises small nuclear ribonucleoprotein (snRNP) complexes (U1, U2, U4/U6, and U5 snRNP) that carry functional uridine-rich small nuclear RNAs (U1, U2, U4/U6, and U5 snRNA), respectively [14], together with over 50 intrinsic proteins [15, 16]. It also contains specific extrinsic non-spliceosomal RNA-binding proteins that regulate protein-RNA crosslink sites and splicing, such as the canonical heterogeneous nuclear ribonucleoproteins (hnRNPs) [17], serine-arginine amino acid-rich proteins (SR proteins) [18] and other tissue-specific splicing factors [19]. The intrinsic complexes and regulatory proteins act as trans-regulation factors of RNA splicing.

**Fig. 1:**
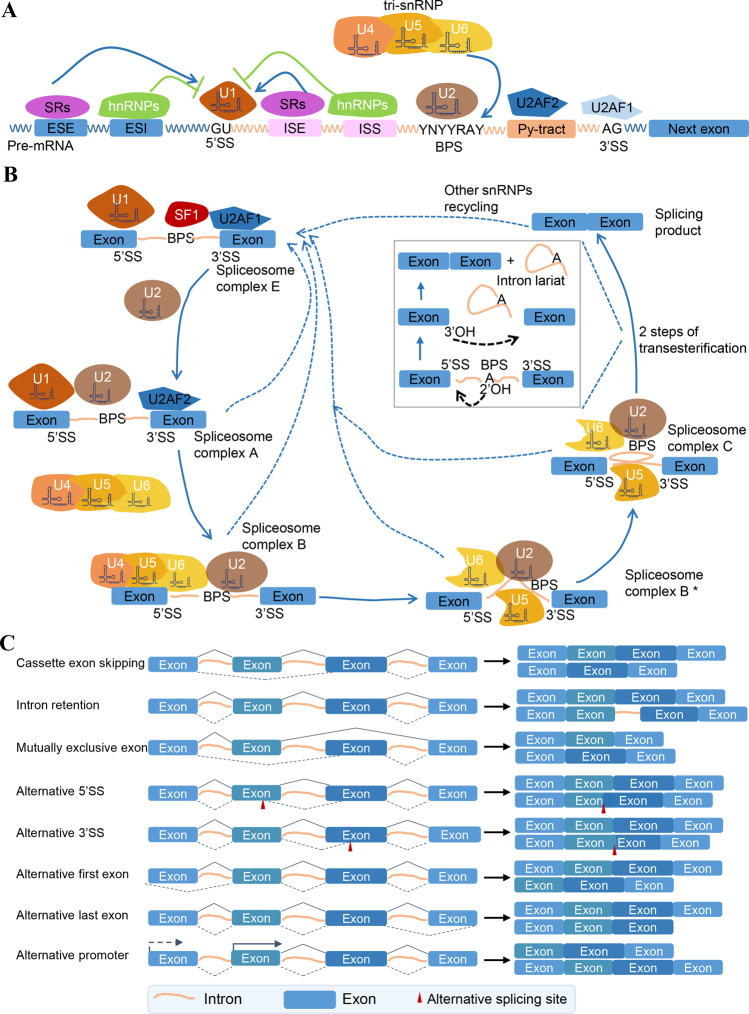
Components of the splicesome machinery and the common types of alternative splicing. The figure shows an outline diagram of splicesome components and clinical treatment targets with experimental evidence. **A** The elements that participate in pre-mRNA splicing. The U1, U2, U3, U4, and U5 are small nuclear ribonucleoprotein (snRNP) complexes that directly bind to splicing sites by recognization between snRNAs and pre-mRNA. The 5’ splice site (5’ SS), the branch point sequence (BPS) and the 3’ splice site (3’ SS) are relatively conserved sequences recognized by snRNPs. The major spliceosome splices introns containing GU at the 5’ SS and AG at the 3’ SS. The typical sequence of BPS is YNYYRAY (Y: pyrimidine; N: any nucleotide; R: purine; A: adenine). The classical/canonical hnRNPs (Heterogeneous nuclear ribonucleoproteins) and SR proteins (serine/arginine amino acid-rich proteins) regulate splicing by binding to the splicing cis-regulatory elements, including exonic splicing enhancer (ESE), exonic splicing silencer (ESS), intronic splicing enhancer (ISE), and intronic splicing silencer (ISS) sequences. **B** The major splicing process is accompanied by the interaction between cis-elements and trans-factors with spliceosome assembly cycle. The gray box shows the stepwise interaction of the small nuclear ribonucleoprotein (snRNP) particles changes with the removal of an intron from a pre-mRNA. The first step of splicing is trans-elements bind to the conserved sequence of introns, including U1 binding to 5’SS, SF1 binding to BPS and U2AF2 binding to Py-tract and U2AD1 binding to 3’SS, which forms the first spliceosome complex E. Then U2 will replace SF1 and interact with BPS, forming the spliceosome complex A. And U4/U5/U6 tri-snRNP substitutes U1, with U5 binding to 5’SS and U6 binding to U2. After that, U4 dissociates from the B complex and some regulatory splicing proteins are recruited, forming the early B act complex (B*). Two steps of transesterification complete splicing progress. U6/U2 catalyzes transesterification reactions by making the BPS ligate to 5’-end of the intron and form a lariat, and the 5’ site is cleaved, resulting in the formation of the lariat. This is followed by a 5ʹSS-mediated attack on the 3ʹSS, leading to the removal of the intron lariat and the formation of the spliced RNA product. The proteins are recycled and used in the next splicing process (showed as dotted lines). **C** Common models of alternative splicing and the corresponding transcript variants. The solid and dashed lines denote different alternative splicing models. Cassette exon skipping: an intervening exon between two other exons can be either included or skipped. Intron retention: an intron remains in the mature mRNA instead of being spliced. Mutually exclusive exon: only one out of two exons (or one group out of two exon groups) is retained with the other one is spliced out. Alternative 5’SS: a potential 5’SS replaces the consensus 5’SS and is joined to 3’SS. Alternative 3’SS: a potential 3’SS replaces the consensus 3’SS and is joined to 5’SS. Alternative first exon: the first exon is replaced by the identical boundaries in the second exon and is exclusive. Alternative last exon: the last exon is substituted by the penultimate with a similar splicing site and exclusive. Alternative promoter: alternative transcription initiation sites also affect the splicing pattern of downstream exons.

The cis-regulatory elements consist of direct binding sites and indirect regulatory sites that participate in fundamental and complex steps of pre-mRNA splicing modification (Fig. 1A). Three consensus sequence elements directly bind to trans-regulatory factors and promote the transesterification reactions of splicing, including the 5’ splice site (5’ SS, also called donor site), branch point sequence (BPS) and 3’ splice site (3’ SS, also called acceptor site) [20]. Certain cis-regulatory elements that occur in exonic splicing enhancer (ESE), exonic splicing silencer (ESS), intronic splicing enhancer (ISE), and intronic splicing silencer (ISS) sequences are crucial elements in the regulation of splicing [21]. Overall, SR proteins recognize and bind to splicing enhancer elements (ESEs and ISEs), whereas hnRNPs target splicing silencer elements [22].

The main splicing process, which includes several key mechanisms, such as RNA–protein interactions, splicing factors with ESE, ESS, IES, and ISS interactions, RNA–RNA base-pairing interactions, and chromatin-based effects, is accompanied by spliceosome assembly, activity, and disassembly cycle (Fig. 1B) [22]. The initial splicing procedure begins with recognition of the 5’ SS GU ribonucleotide by U1 snRNP and recognition of the branch point sequence (BPS) by Splicing Factor 1 (SF1) that forms the first spliceosome complex E. The U2 Auxiliary Factor 2 (U2AF2) binds to polypyrimidine tract (Py-tract) or U2 Auxiliary Factor 1 (U2AF1) binds to 3’ SS [23]. Afterward, U2 snRNP and the preassembled U4/U6/U5 tri-snRNP occupy and then are substituted in turns among splicing process, and final transesterification reactions form a mature mRNA with a lariat (detail illustrated in Fig. 1B) [24–26].

### The common types of alternative splicing

Alternative sites that possess similar sequences to the consensus splice elements are spliced by spliceosome to generate a variety of mature mRNAs because of attenuated affinity between consensus splice site and snRNPs. High-throughput RNA sequencing and analysis revealed that 90–95% of human multi-exon genes produce transcript variants through AS [27]. The common types of AS include cassette exon skipping, intron retention, mutually exclusive exon, alternative 5’ splice site, alternative 3’ splice site, alternative first and last exon (AFE and ALE, respectively), and alternative promoters (Fig. 1C). Moreover, cassette exon skipping and intron retention are predominant in human AS types [5, 28]. Variable AS events not only produce thousands of variants, but also exhibit tissue or pathological specificity, and provide new targets for diagnosis and treatment of cancer [5, 29].

## Aberrant regulation of alternative splicing associated with CRC

**Fig. 2:**
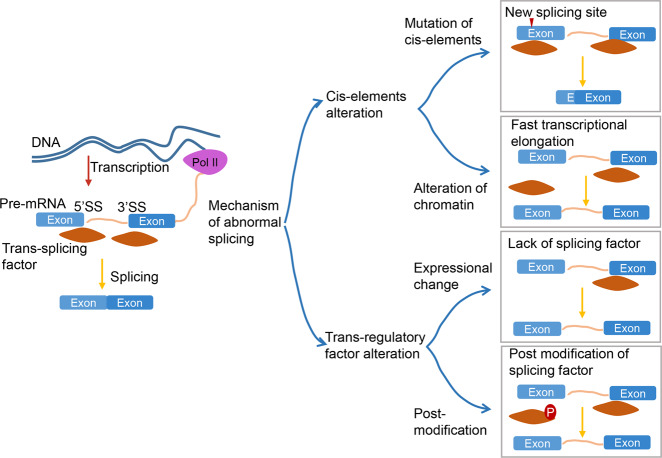
The mechanism of aberrant splicing in colorectal cancer. The diagram shows the classifiable explanation of abnormal splicing in CRC. Splicing occurs co-transcriptionally on nascent RNA, which is attached to chromatin by RNA polymerase II (showed in the left figure). Both alterations of cis-elements and trans-regulatory factors would cause abnormal splicing events and products. New recognized sites are created by mutation of cis-elements like single base substitutions, translocation, and alternative promoters (the first diagram on the right). Alteration of chromatin would influence affinity between splicing factors to splicing sites by conformational change or speed of transcription elongation with changed time of splicing factors loading on cis-elements (the second diagram on the right). In addition, the expression and post-modification of trans-regulatory alter the splicing by infecting recognization between splicing factors and splicing sites.

Aberrant AS has resulted in great achievements in CRC treatment with the advancement in sequencing technology, such as a cohort study of 1231 CRC cases with targeting sequencing of 36 putative CRC susceptibility genes, revealed that 8 genes of them generated 36 novel specific protein isoforms due to AS [30]. The dysregulation mechanism can be ascribed to two common aspects: cis-elements and trans-regulation factors (Fig. 2). The reasons for aberrant AS and the generation of corresponding transcript variants in CRC are discussed in the subsequent section.

### Variable cis-regulatory elements

The cis-element sequence and structure contribute to the regulation of AS. Generally, the velocity of splicing is dependent on the speed of splicing machineries and nascent pre-mRNA transcription elongation when splicing occurs co-transcriptionally following the translocation of RNA polymerase II (Pol II) [31]. Pre-mRNA sequences carry splicing codes and influence protein function, whereas chromatin structure like histone nail modification and Pol II transcription influence splicing by adjusting the affinity of cis-elements to trans-regulatory factors and speed of transcription [32]. The effect of cis-elements changes on AS can be classified into three categories: single-base substitutions, translocation, and alteration of chromatin or promoters. Additional aberrant cis-regulation mechanisms are listed in Table 1.

**Table 1:** The aberrant changes of cis element of splicing.

Gene	Mutant site	Mutant type	Splicing mechanism	Splicing alteration	Somatic or germline	Reference
BRCA2	c.7802A>G	Single base substitution	Splice donor site	Skipping the last four nucleotides of exon 16	Germline mutation	[127]
BRF1	c.1459+2T>C	Single base substitution	Splice donor site	Skipping of exon 13	Germline mutation	[38]
DPDY	c.321+2T>C	Single base substitution	Splice donor site	Skipping of exon 4	–	[128]
MLH1	c.543C>T	Single base substitution	Splice donor site	Deletion of the last 4 bases of exon 6	Germline mutation	[129]
BRCA2	c.67+3A>G	Single base substitution	Splice donor site	Skipping of exon 2	Germline mutation	[130]
MSH2	c.2634+1G>C	Single base substitution	–	Skipping of exon 15	Germline mutation	[131]
	c.212-1G>A	Single base substitution	Splice acceptor site	Deletion 80-85 bases of exon 2	Germline mutation	[37]
PMS2	c.2002A>G	Single base substitution	Splice donor site at intron 11	5 bp deletion at the exon 11–12 junction	Germline mutation	[39]
TP53	c.96+1G>A	Single base substitution	Splice donor site	Exon 3 skipping	Somatic mutation	[132]
	c.782+1G>A	Single base substitution	Splice donor site	Intron 7 retention	Somatic mutation	
	c.920–2A>G	Single base substitution	Splice acceptor site	Intron 8 retention or exon 9 skipping	Somatic mutation	
	c.919+1G>A	Single base substitution	Splice donor site	Intron 8 retention or exon 8 skipping	Somatic mutation	
	c.560–1G>A	Single base substitution	Splice acceptor site	Intron 5 retention or exon 6 skipping	Somatic mutation	
MSH2	MSH2 (hg19, chr2: 47 653 051)	Invertion of AluY repeat in intron1,6	Donor site at exon1, acceptor site at exon7	Skipping of exon2-6	Germline mutation	[41]
	c.2635-3delC	Single base missing	Acceptor site of exon 16	Part of intron 15 retention	Germline mutation	[42]
MLH1	c.545+4545+5delCA	Deletion of 2 bp in splice donor site	Donor site of exon 6	Skipping of exon 6	Germline mutation	[43]
APC	c.1226-1229delTTTTinsAAA	Insertion–deletion at codon 409	5’ end of exon 9	Skipping of exon 9	Germline mutation	[44]
HNF4a	–	Alterantive promoter	Different pre-mRNA	Skipping of exon 1	–	[47, 48]
MCL1	–	Histone acetylation	Increase H3/4 acetylation	Skiping of exon2	–	[45]
DVL	–	Histone methyation	Decrease H3K36 methylation	Intron 2 retention	–	[46]

#### Single base substitution

The integrated analyses of DNA and RNA sequences revealed that somatic mutations at donor sites or acceptor sites can increase the potential of aberrant splicing events and new transcript variants [33, 34]. Single base substitution can adequately induce aberrant splicing events in patients with CRC [35], especially when mutations occur at direct splice sites or regulatory elements. A study of 369 patients with Lynch syndrome cohort revealed that ~40% of patients carried the mutL homolog 1 (MLH1) mutation, with the most common type of mutation was direct splice site alterations [36], which was consistent with the findings of computational analyses conducted by Frey [32].

Specific single base mutations could create a new splicing site as a competitor, such as mutations at the acceptor splice site of MSH2 (c.212-1G>A), which induces activation of a new splice site in exon 2 and generates truncated proteins (p.Gly71Valfs*2 and p.Gly71Glufs*75), is identified in young patients with genetic predisposition to colon cancer [37]. In addition, single base substitution with offering novel splicing sites of other genes was presented in Table 1, such as BRF1 RNA polymerase III transcription initiation factor subunit (BRF1) germline mutation of c.1459+2T>C [38], PMS1 homolog 2 (PMS2) mutation of c.2002A>G [39].

Furthermore, loci of the mutations in ESE, ESS, IES, or ISS elements are also key splicing regulators in CRC. The SNPs of ISS sites between exon4 and 5 in O-GlcNAc transferase (OGT) can increase mRNA intron retention variants by slowing down splicing speed, which induces tumorigenesis [40]. Although numerous studies on germline mutations are associated with AS in CRC, few studies have reported the mechanisms of somatic mutations in patients with CRC clearly.

#### Translocation

Translocation of sequences, including insertion or deletion of a long or short gene fragment, is a primary regulatory mechanism of splicing. These phenomena have been observed in patients with Lynch syndrome, an inherited condition that increases the risk of colon cancer. Single base-pair deletion, MSH2 (c.2635-3delC) lost the ability of the splicing acceptor site promotes an aberrant variant with intron 15 retention, whereas paracentric inversion AluY repeats brings novel splicing donor site and acceptor site at exon 1 and exon 7, and then generates a truncated isoform lacking exon2-6 [41, 42]. Details of other translocations that are related to splicing in CRC are shown in Table 1, such as germline mutation of 2 base-pair deletions at the splice donor site of MLH1 exon 6 [43] and insertion-deletion (indel) at codon 409 of APC [44].

#### Aberrant chromatin and promoters

Splicing events are influenced by the chromatin features, such as epigenetic modification of DNA methylation and histone acetylation. In HCT 116 cells, inhibition of histone deacetylase (HDAC) that specifically increases K (lysine) acetyltransferase 2B (KAT2B) occupancy and histone3/4 acetylation nucleosome can increase exon 2 exclusion in myeloid cell leukemia sequence 1 (MCL1) transcript variant [45]. Inhibition of HDAC resulted in hyperacetylation of H3K4me3 nucleosomes and increased the rate of elongation through these regions, which did not leave sufficient time for the loading of SRSF1 onto exon 2. The absence of SRSF1 and other splicing factors at exon 2 resulted in increasing the MCL1 exon 2 exclusion [45]. And the trimethylation of H3K36me3 accelerated transcription elongation and reduced the removal of intron 2 of disheveled segment polarity protein 2 (DVL2) [46]. In addition, different transcriptional initiation sites can alter splicing with additional splicing sequences. The prominent promoter 2 of hepatocyte nuclear factor 4 alpha (HNF4a), rather than promoter1, is transcribed into P2-HNF4a pre-mRNA with extra exon 1D and provides more splicing sites, which results in increased pro-proliferation HNF4a7/8 splice variants [47, 48].

### Alterations of splicing trans-regulatory factors

In CRC, alternative splicing can be regulated by trans-regulatory factors in form of mutations, dysregulated expressions or protein-modifications of RNA-binding proteins (RBPs) that occur in spliceosome components and splicing regulators [49, 50]. Alterations that occur in trans-regulatory factors are shown in Table 2.

**Table 2:** The aberrant change of trans-regulation splicing factors in colorectal cancer.

Trans-regulation factor	Classification	Alteration	Aberrant mechanism	Splicing target	Product change	Splicing effect	Referrence
SRSF1	SRs	Upregulated	Transcription upregulation	DBF4B	Full-length DBF4B upregulated	DBF4B exon6 inclusion	[54]
SRSF1	SRs	Upregulated	Transcription upregulation	HNRPLL	HNRPLL-E12A RNA upregulated	HNRPLL with Exon12a	[55]
SRSF3	SRs	Upregulated	–	PDCD4	PDCD4 AS-1 upregulated	Part of exons 2 and 3 skipping	[56]
SRSF3	SRs	Upregulated	Transcription upregulation	PKM	PKM1/PKM2 upregulated	PKM1(exon 9 inclusion)	[57]
PTBP1	hnRNPs	Upregulated	Transcription upregulation	PKM	PKM1/PKM2 downregulated	PKM2(exon 10 inclusion)	[57, 63–66, 133]
hnRNPA1	hnRNPs	Upregulated	Transcription upregulation	PKM	PKM1/PKM2 upregulated		[57]
SRSF3	SRs	Upregulated	Protein stability upregulation	MAP4K4	Isoforms 2 and 5 upregulated	Exon16 inclusion	[58]
SRSF6	SRs	Upregulated	Transcription upregulation	ZO-1	ZO-1 E23− (exon23 skipping) variants upregulated	Exon 23 skipping	[59]
SRSF7	SRs	Upregulated	Transcription upregulation	FAS	FAS-S: short isoform of exon 6 skipping	Exon 6 skipping	[60]
SRSF10	SRs	Upregulated	Transcription upregulation	BCLAF1	BCLAF1-L(exon5a inclusion) upregulated	Exon5a inclusion	[61]
hnRNPK	hnRNPs	Upregulated	Transcription upregulation	MRPL33	MRPL33-long isoform upregulation	Exon 3 inclusion	[62]
hnRNPLL	hnRNPs	Downregulated	Transcriptiondownregulation	CD44	Cd44v6 (variable exon 6) upregulated	Exon v6 inclusion	[96]
PTBP1	hnRNPs	Upregulated	Transcription upregulation	RAC1	RAC1 isoform b upregulated	Exon 3b inclusion	[64]
PTBP1	hnRNPs	Upregulated	Transcription upregulation	NUMB	NUMB Isoform 1 upregulated	Exon 9 inclusion	[64]
ESRP2	Other splicing regulator	Upregulated	Transcription upregulation	ITGA6	ITGA6A upregulated	Exon 25 skipping	[115]
EFTUD2	spliceosome	Upregulated	Transcription upregulation	MyD88	MYD88 long isoform upregulated	Full-length (containing intermediate domain)	[52]
EFTUD2	spliceosome	Upregulated	Transcription upregulation	MD-2	MD-2A long isoform upregulated	Full-length (contain the first 54 bases of exon 3)	[52]
RBM4	Other splicing regulator	Downregulated	Transcription downregulation	PTB	nPTB upregulated	Exon 10 inclusion	[68]
RBM4	Other splicing regulator	Downregulated	Transcription downregulation	NOVA1	Nova1(-4) upregulated	Exon 4 skipping	[69]
TRA2β, SRSF1	SRs	Upregulated	Transcription upregulation	BCL2	BCL2α upregulated	Exon 3 skipping	[77]
PRPF6	spliceosome	Upregulated	Transcription upregulation	ZAK	ZAK-LF isoform upregulated	Exon 12 inclusion	[134]
hnRNP L	hnRNPs	Upregulated	Transcription upregulation	CEACAM1	CEACAM1-L upregulated	Exon 7 inclusion	[93]
hnRNP A1	hnRNPs	HOXB-AS3 peptide binds to hnRNP A1	Decreasing HOXB-AS3 binding to hnRNP A1	PKM	PKM2 upregulated	Exon 10 inclusion	[72]
hnRNPA1	hnRNPs	Phosphorylation of hnRNPA1 upregulated	Phosphorylation of Ser6 of hnRNPA1 enhanced by S6K2	PKM	PKM2 upregulated	Exon 10 inclusion	[71]
PHF5A	spliceosome	PHF5A K29 acetylation	PHF5A K29 acetylation increased by p300	KDM3A	Intron3 retetion KDM3A variant downregulated	Intron 3 retention	[70]

#### Altered expressions of trans-regulatory factors

Alterations in expression levels of spliceosome components and splicing regulatory proteins are attributed to transcriptional or posttranscriptional regulation, thereby controlling various aberrant splicing events that are closely associated with CRC progression (Fig. 2; Table 2). Splicing factor 3b subunit 1 (SF3B1), a core spliceosome component that causes more than 2000 AS events, is associated with most intron retention in CRC cells when it is downregulated [51]. The elongation factor Tu GTP binding domain containing 2 (EFTUD2), a component of the U5 snRNP, is upregulated in colitis-associated cancer. EFTUD2 promotes colitis-associated tumorigenesis by splicing regulation of increased proportion of full-length variant of innate immune signal transduction adaptor (MyD88), since the complete U5 is required for retention of exon 2 [52, 53].

Serine and arginine-rich splicing factor 1 (SRSF1) is a key member of SRs family. It is an oncogenic factor that promotes tumorigenesis by regulating various AS events, such as splicing of Rac Family Small GTPase 1 (RAC1), Tyrosine-protein kinase (SYK), Marker of proliferation Ki-67(MKI67) and heterogeneous nuclear ribonucleoprotein L like (HNRPLL) [51, 54, 55]. In CRC, SRSF1 has been found to be upregulated and is associated with DNA damage and cell cycle progression [50]. Elevated SRSF1 binds 3’SS of DBF4B exon 6 in colon cancer cells to activate it [54, 55]. Other splicing factors, such as SRSF3, SRSF6, SRSF7 and SRSF10 also regulate the splicing of different targets in CRC as oncogenes [56–61].

HnRNPs are primary splicing regulation factors that bind to ESS and ISS elements. Heterogeneous nuclear ribonucleoprotein K (HnRNPK) recognizes exon 3 of mitochondrial ribosomal protein L33 (MRPL33) pre-mRNA. It upregulates MRPL33-L, an exon 3-containing long isoform, through which it exerts its role in maintaining tumorigenic phenotypes of the colon [62].

Other splicing regulatory factors, such as RNA-binding motif protein 4 (RBM4), whose expression is suppressed in cancer, is a tumor suppressor that dysregulates exon 4 skipping of NOVA alternative splicing regulator 1 (NOVA1) and intron 11 retention of PTB [58]. PTBP1 is upregulated by a more stable variant with intron 11 retention and the upregulated PTBP1 is a marker of poor survival outcomes [57, 63]. PTBP1 increases the ratio of the PKM2/PKM1 variant by binding intron 8 of PKM and elevating the PKM2 transcript variant with exon 9 skipping [64–66], thereby enhancing the Warburg effect and promoting tumor progression [67–69].

#### Posttranslational alterations of trans-regulatory splicing factors

Protein modifications can modulate the splicing ability of trans-regulatory splicing factors (Fig. 2; Table 2). PHD finger protein 5A (PHF5A), a component of U2 snRNPs complex, can be acetylated at lysine 29 by p300 to strengthen interactions among components of U2 snRNPs during colorectal tumorigenesis. The tight complex reduces the retention of intron 3 variant of KDM3A, which triggers the degradation of abnormal mRNAs with early stop codons [70]. Additionally, PHF5A-K29 acetylation is s poor prognostic marker for 3-year overall survival rate [70]. Phosphorylation of the hnRNP A1 Ser6 site by the S6K2 enzyme facilitates the binding of hnRNPA1 to the splicing site of the PKM gene to enhance the generation of PKM2 variants in CRC [71].

Protein interactions between splicing factors regulate splicing by blocking RNA-protein or spliceosome-protein interactions (Fig. 2; Table 2). The HOXB-AS3 peptide competitively binds arginine residues in the RGG motif of hnRNP A1, a splicing regulatory factor that promotes PKM2 variant by flanking 5’SS of exon 9, and thereby excluding exon 9. In CRC cells, HOXB-AS3 has been found to be downregulated, and subsequently, causes PKM2 upregulation that leads to metabolic disorders by antagonizing hnRNP A1 recognition of PKM exon 9 [72].

## Alternative splicing in CRC progression

**Fig. 3:**
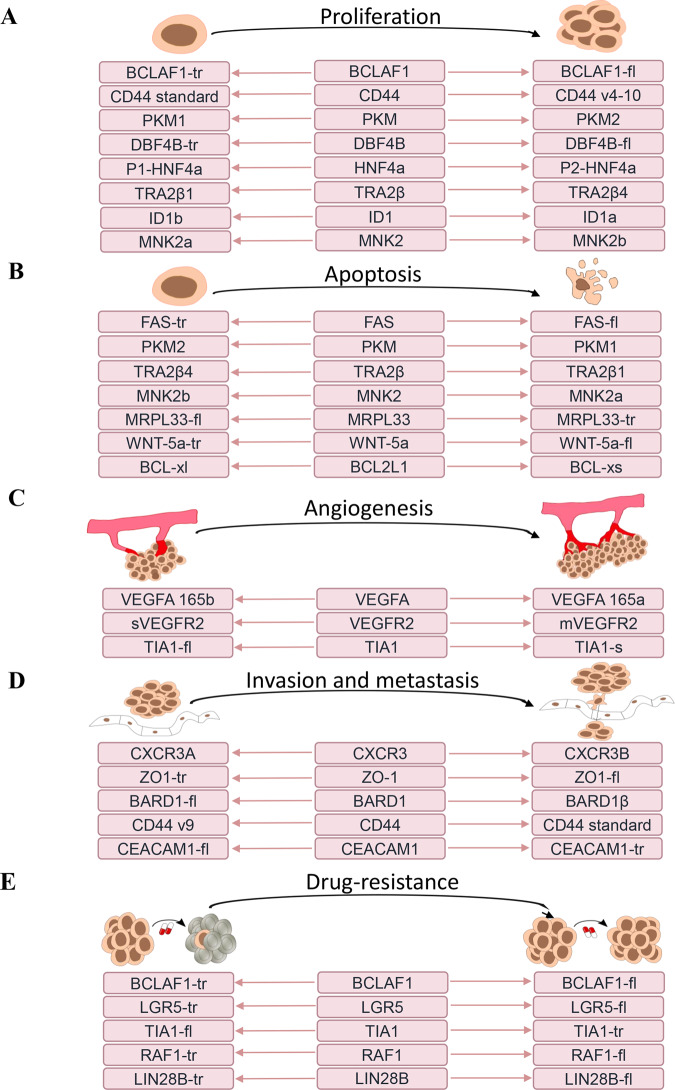
Alternative splicing is associated with tumor hallmarks. Five common tumor hallmarks related to alternative splicing include proliferation, invasion and migration, apoptosis, angiogenesis, and drug resistance. The figure shows the hallmarks and the associated genes. **A** The representative gene and their splicing variants show different functions in cell proliferation. **B** The representative gene and their splicing variants show different functions in cell apoptosis. **C** The representative gene and their splicing variants show different functions in angiogenesis. **D** The representative gene and their splicing variants show different functions in invasion and metastasis. **E** The representative gene and their splicing variants show different functions in drug resistance. More details about the mechanism of splicing and clinical application are listed in Supplementary Table 1.

AS is involved in CRC progression and it plays a key role in multiple malignant hallmarks, including sustaining proliferation, inhibiting apoptosis, angiogenesis, aberrant metabolism, invasion and metastasis. We reviewed the splicing variants that are strongly associated with the malignant hallmarks of CRC, and highlighted the variants with clinical relevance (Fig. 3A–E; Supplementary Table 1). And some gene variants in CRC have several malignant functions (Supplementary Table 1) are discussed with their main malignant function. For example, PKM2 variants promote proliferation and inhibit apoptosis, the CD44 v4-10 variant promotes proliferation, the CD44 v6 variant promotes invasion, the CD44 v9 variant inhibits invasion while the CD44 s variant inhibits proliferation. PKM variants are majorly involved in proliferation while CD44 variants are involved in both proliferation and invasion.

### Sustaining proliferation

Sustaining proliferation is one of the malignant features of CRC cells and tumor growth, which can be promoted through the regulation of splicing with additional oncogenic splicing isoforms. The CD44 gene is composed of 20 exons, the first exons 1–5 and the last exons 16–20 are constant exons that encode for the N-terminal and C-terminal regions of CD44, respectively. Middle variable exons, respectively abbreviated as exons v1-v10, can be alternatively spliced and translated into different isoforms [73]. Elevated expression of the CD44 v4-10 isoform, which comprises constant and v4-v10 exons, can respond to HGF and Wnt-mediated signals through interactions between the v6 exon and HGF, thereby promoting tumor cell proliferation by maintaining the stemness of cancer stem cells, a feature that is not exhibited by CD44s [74, 75]. This leads to poorer survival rates as was observed in transgenic Cd44^v4-10/v4-10^ mice, when compared to CD44^s/s^ mice [75].

The PKM protein has a switching effect on a specific glycolysis metabolism. Classical splicing variants, PKM1 and PKM2 contain exon 9 or exon 10 (with mutually exclusive exon), respectively, which are under the regulation of hnRNPA1, PTBP1, and SRSF3 [57]. When compared to normal epithelial cells, PKM2 is upregulated by hnRNPA1 overexpression or activation of phosphorylation, which facilitates the binding of hnRNPA1 to the splicing site [57, 71]. Elevated expressions of PTBP1 upregulate the PKM2 variant with exon 9 skipping (mentioned in section “Altered expressions of trans-regulatory factors”) [64–66]. Elevated levels of PKM2 promote aberrant metabolism by increasing glycolysis and lactate generation rather than the TCA cycle regardless of oxygen availability, which in turn, accelerates cell proliferation and colon cancer progression [72]. Clinically, univariate and multivariate Cox regression analyses revealed that the expression of both PTBP1 and PKM2 is a significant risk factor for the overall survival of patients with CRC [57, 63, 76].

A full-length variant containing DBF4B exon 6, which has been found to be upregulated in tumors, binds SRSF1 to recognize the 3’ SS of DBF4B. Both DBF4B full-length (DBF4B-FL) variant and SRSF1 promote tumor cell proliferation and tumor growth while the DBF4B-short (DBF4B-S) variant does not exhibit a similar function. Moreover, patients with a low DBF4B FL/S ratios have significantly longer median survival outcomes than patients with increased DBF4B FL/S expression ratios [54]. Figure 3A shows some of the proliferation-related gene variants, including HNF4a [47, 48], TRA2β [77], and ID1 [78] with a detailed description in Supplementary Table 1.

### Apoptosis

A typical characteristic of malignant tumors is evasion of apoptosis, which is responsible for tumor formation and maintenance. The full-length Fas cell surface death receptor (FAS) protein, also referred to as CD95, is located on the cytomembrane as a receptor. It initiates a cascade of events that eventually lead to programmed cell death. Exon 6 of Fas encodes a transmembrane domain, but skipping of the exon through regulation of SRSF7 splicing produces an mRNA encoding a soluble Fas isoform called Fas-short. SRSF7 is upregulated in CRC cells and it increases the ratio of Fas-short variant, resulting in the loss of function of apoptosis induction, thereby exhibiting the inverse feature of the Fas full-length variant [60, 79].

BCL2 Like 1 (BCL2L1), a member of BCL2 Apoptosis Regulator (BCL2) family, is spliced into two isoforms when regulated by hnRNPA2B1 [80]. The BCL-xl isoform of BCL2L1 is 233 amino acids in length and exhibits anti-apoptotic effects, while BCL-xs, which lacks 63 amino acids in exon 2 compared to BCL-xl, is pro-apoptotic [81]. The aberrance of hnRNPA2B1 can result in a switch in the BCL2L1 gene from BCL-xl to BCL-xs in CRC [80]. In addition, BCL2 Associated X (BAX), another key member of the BCL2 family, has been found to have a pro-apoptotic variant (Bax∆2) with exon 2 skipping. Bax∆2 triggers apoptosis through a non-mitochondrial pathway, while the Baxα variant promotes mitochondria-dependent apoptosis [82, 83].

MAPK interacting serine/threonine kinase 2 (MNK2) can be spliced by SRSF1 to generate two types of isoforms, MNK2a and MNK2b. MNK2a comprises exon 14a, whereas MNK2b lacks exon 14a. In CRC cells, an imbalance exists between the two isoforms because due to elevated SRSF1 expression, MNK2b is dominant while the MNK2a isoform, which has a MAPK-binding domain, is downregulated, thereby promoting cell growth and reducing cell apoptosis by inhibiting the p38a-MAPK signaling pathway [84].

### Angiogenesis

The clinical outcome of patients with CRC is strongly correlated with angiogenesis, which is stimulated by the vascular endothelial growth factor-A (VEGF-A) [85]. There are 7 major types of AS isoforms of VEGF (angiogenic VEGF-xxxa and antiangiogenic VEGF-xxxb) generated by exon skipping in CRC [85–87]. The most studied isoforms are VEGF165a and VEGF165b, which are regulated by SRSF6 and exhibit reversed functions in angiogenesis. VEGF165a is upregulated in CRC and promotes angiogenesis, vessel maturation and cell migration [69]. However, VEGF165b is antiangiogenic and most studies have revealed that it is a favorable prognostic factor in patients with CRC, except for a study by Kotoula that revealed that VEGF165b was associated with poor prognosis in patients with right-sided primary tumors [85, 88–90]. In addition, VEGF receptors (VEGFRs) are alternatively spliced and can regulate angiogenesis; for example, VEGFR2 is expressed as two isoforms, membrane-bound VEGFR2 (mVEGFR2) and soluble VEGFR2 (sVEGFR2). VEGF promotes angiogenesis by binding to membrane-bound vascular endothelial growth factor receptor 2 (mVEGFR2), whereas sVEGFR2 sequesters VEGF and is thus anti-angiogenic [91].

### Invasion and metastasis

Antigen cell adhesion molecule 1 (CEACAM1) is a member of the carcinoembryonic antigen (CEA) family that function as an intercellular adhesion molecule that influence the recurrence of colorectal liver metastasis after hepatectomy [92, 93]. AS of exon 7 by hnRNPL generates two variants, CEACAM1-long and CEACAM1-short (without exon 7). In invasive tumor cells, there is a high proportion of the CEACAM1-short variant, which promotes migration, invasion and proliferation, functions which are not performed by CEACAM1-long [94, 95]. The high expression of CEACAM1-S enhances tumor-initiating of CRC in a metastasis induction model and is negatively correlated with five-year recurrence-free survival rates, and overall survival rates of patients with CRC [92].

Furthermore, CD44 v6 is another CD44 splicing variant, which contains v6 exons by HNRNPLL splicing regulation, is highly expressed in CRC [96]. CD44 v6 isoform can enhance EMT progress, cell motility and invasion as an intercellular communicator when located on the cytomembrane or exosome [97, 98]. The high levels of CD44v6 can be used as a marker for predicting poor prognosis in stage II and stage III sporadic CRC compared with CD44s. In addition, high expression of CD44v6 is significantly correlated with poor histological differentiation, deeper tumor invasion, increased lymph node metastases, angiolymphatic invasion, and advanced pathological TNM (Tumor, Node, Metastasis) staging in the clinical-pathological analysis of CRC [99].

### Drug resistance

AS has been reported to influence the development of drug resistance in malignant cancer cells. BCL-2-associated X (BAX) is an apoptosis regulator that can be spliced into a unique Bax isoform (Bax∆2) in colon cancer cells because of BAX microsatellite G7/G7 alleles [83, 100]. Bax∆2-positive cells can recruit caspase-8 into the proximity for activation, and subsequently activate caspase-3 and apoptosis independent of the mitochondrial pathway [83]. Overexpression of Bax∆2 can enhance sensitivity of tumor cells to proteasome inhibitors, such as bortezomib and carfilzomib, which could be a novel chemotherapeutic target for cancer treatment [101].

Bevacizumab, normally recommended for the treatment of patients with metastatic CRC patients with metastasis, targets VEGF signaling. Bevacizumab response is associated with AS of VEGF isoforms. For example, the VEGFA145b isoform may predict resistance to bevacizumab in patients with left-sided primary CRC [85]. Results of immunohistochemical analyses of CRC have suggested that plasma VEGF-Axxxb levels could be an effective biomarker of response to bevacizumab [90, 102]. Previous studies also revealed that xenograft mice with a high ratio of VEGF165b isoform were more sensitive to bevacizumab treatment and hence had tumors with smaller volumes [89].

## Potential tumor diagnosis and treatment targets for CRC

Aberrant splicing mechanisms and the corresponding products are potential diagnostic markers and predictors of the survival of the patients with CRC. The RAS family is significant for CRC progression with several alternative splice variants, which are associated with several clinicopathological features of CRC [103]. K-RAS4A, retaining extra exon 4A when comparing with K-RAS4B, is associated with superior 5-year overall survival in KRAS wild-type subgroup and clinicopathological features of left colon, adenocarcinoma subtype. Nonetheless, KRAS4B overexpression is significantly associated with larger tumor size and inversely correlated with p27kip1 protein [104–106]. Beyond that, some other variants, such as ZO-1 exon23 skipping isoform, CD44v6, CEACAM1-Short isoform, Δ133p53β with inron4 retention were negative survival markers in CRC [59, 92, 97, 98]. The VEGFA 165b and high ratio of CD44v9/CD44s predicted good prognosis of patients with CRC [85, 88–90](Supplementary Table 1).

**Fig. 4:**
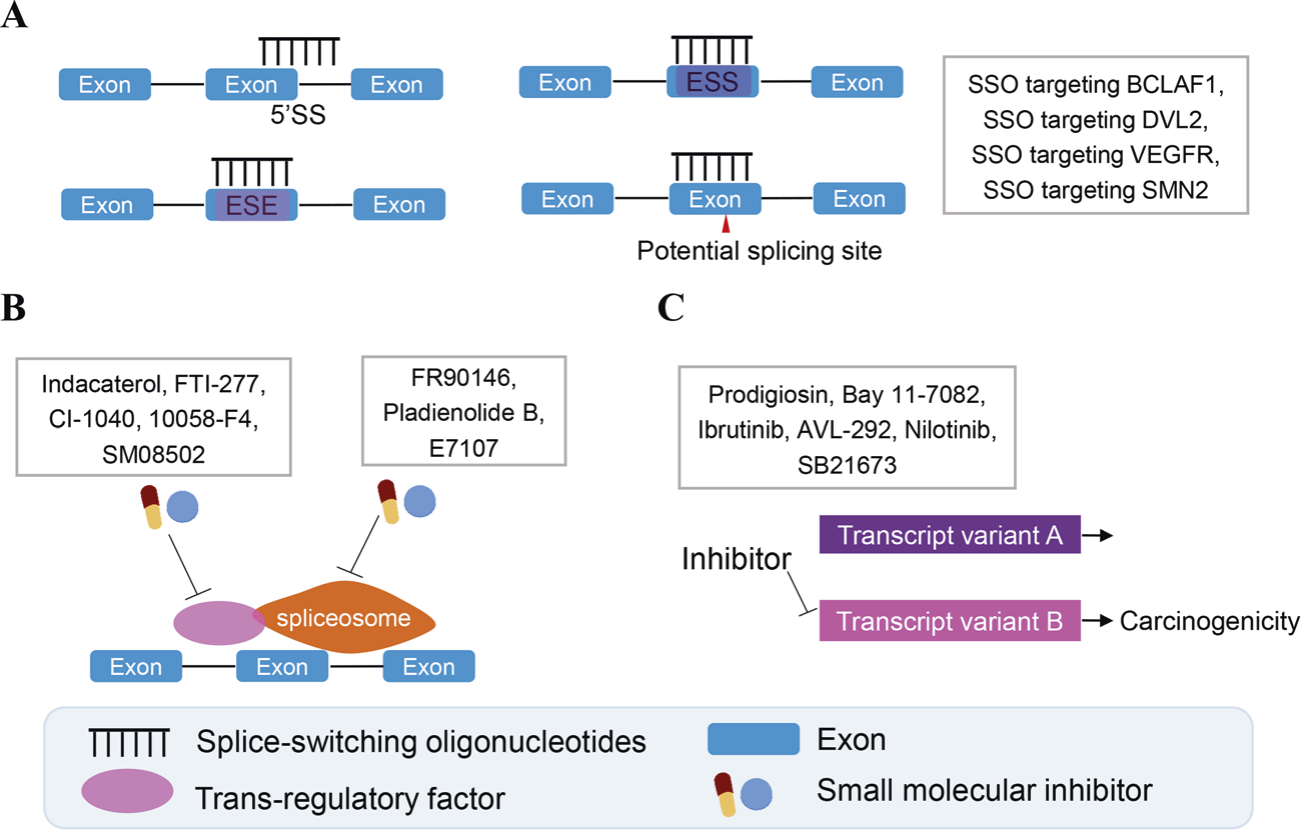
The potential therapeutic strategies for treating patients with CRC by splicing alterations. Therapeutic strategies of splicing alterations include both the direct nucleic acid sites and splicing regulatory factor. **A** An ideogram shows splice-switching oligonucleotides (SSOs) targeting direct splicing site (5’SS or 3’SS), exon splicing enhancer (ESE) or inhibitor (ESI) and potential splicing sites. SSOs with experimental evidence were shown in the diagram. See text and Table 3 for detail. **B** Trans-regulatory factors are also targeted by small molecule inhibitors as treatment strategies through the splicing mechanism. Small molecule inhibitors targeting trans-regulatory factors and spliceosome are shown in the diagram. See text and Table 3 for detail. **C** Some special splicing variants related to carcinogenesis are effect targets for inhibitor and colorectal treatment. Inhibitors targeting oncogenic variants or signaling are shown in the diagram. See text and Table 3 for detail.

Additionally, the complex mechanism of AS can provide novel therapeutic targets for patients with CRC. The direct therapeutic strategies of splicing alterations can be divided into 3 categories based on splicing mechanism: (1) strategies targeting cis-elements of splicing, (2) strategies targeting trans-regulatory factors of splicing, including the spliceosome complex and splicing regulation factors, especially the splicing regulation factors, and (3) strategies targeting splicing variants and the downstream mechanisms. Complex splicing mechanisms can be used to develop multiple target therapies for patients with CRC (Fig. 4). The common drugs for splicing intervention are antisense oligonucleotides (ASOs) that target base sequences and small molecules altering the activities of splicing regulatory factors. Agents are listed in Table 3 with experimental evidence to confirm as treatment targets by targeting AS.

**Table 3:** Treatment targets with alternative splicing.

Agents	Splicing gene	Direct targets	Mechanism	Stage of development	Referrence
SSO	BCLAF1	3’SS at the boundary of intron 4 and exon5a	SSO treatment increase truncated isoform against SRSF10 splicing effect and inhibits cell proliferation.	Preclinical Cell level	[61]
SSO	PKM	SSO against exon 10 for PKM	The SSO against exon 10 for PKM gene decreased the mRNA ratio of PKM2/PKM1.	Preclinical Cell level	[59, 71]
SSO	DVL	3′SS of intron 2 of DVL2	The increasing intron2 retention variant for DVL2 can inhibits cell proliferation whether SETD2 exists or not.	Preclinical Cell level	[46]
SSO (2′-OMe phosphorothioation)	HnRNP A1	Blocking 5′ SS of SMN2 exon 7	The SSOs targeted hnRNP A1 binding sites of SMN exon 7 and then reduced oncogenic exon 7 inclusion variant to inhibit CRC.	Preclinical Cell level	[57, 71, 135]
RHPS4, G4 structures	CD133	The 118 bp upstream and 261 bp downstream of acceptor sites of exons 4 and 7	RHPS4 treatment increase intron3,6 retention variant with early termination of translation, then cause more truncated isoform CD133 and surpress cell growth.	Preclinical Cell level Mice level	[121]
Prodigiosin	P73	Upregulated c-Jun, and induced phosphorylation of c-Jun	Prodigiosin induces phosphorylation of c-Jun, which mediates p73 upregulation and ΔNp73 downregulation and then inhibited the growth of xenograft tumors initiation.	Preclinical Cell level Mice level	[117]
Ibrutinib (BTK) AVL-292(BTK)	BTK	BTK kinase inhibitor	The inhibitor of P65BTK oncogeneic isoform depends on p-hnRNPK, which is active by ERK1/2 and RAS.	Preclinical Cell level	[119]
FTI-277 (RAS) CI-1040 (MEK1/2)		Inhibitor of hnRNPK phosphorylation			
Nilotinib (ZAK inhibitor)	ZAK	Inhibitor of ZAK-autophosphorylation and auto-activation	Nilotinib suppresses pro-tumoral reaction cascades of ZAKs, which are key factors in cancer cell migration.	Preclinical Cell level	[118]
Bay 11-7082 (NF-κB inhibitor)	TXL2	NF-κB(downstream of oncogenic TXL-2b variants)	Txl-2b contributes to resistance against vincristine and induces apoptosis by activating NF-κB signaling and blocking the downstream.	Preclinical Cell level	[120]
SSO(locked nucleic acid, LNA)	Aurora-A	Target exon 2-containing Aurora-A 5’-UTR	SSO targets the carcinogenic exon 2-containing Aurora-A mRNA isoforms can inhibit tumor growth.	Preclinical Cell level Mice level	[136]
SSO (antisense morpholino oligomer)	VEGFR	Against 5’SS of exon13-intron13 junction	SSO is directed against the junction sequence that shifts expression from mVEGFR2 to the antiangiogenic sVEGFR.	Preclinical Cell level	[91]
10058-F4(MYC inhibitor)	ITGA6	MYC inhibitor	MYC inhibitor increases the epithelial splicing regulatory protein 2 (ESRP2) and decreases the tumor promoter ITGA6a variant.	Preclinical Cell level Mice level	[115]
SB21673(GSK3β inhibitor)		Inhibiting GSK3β kinase activity	The inhibitor can suppress ITGA6A variant, which interferes with the Wnt/β-catenin pathway by enhancing phosphorylation of β-catenin by GSK3β.		[137]
SM08502 small molecular inhibitor	DVL2, ERBB2, LRP5, TCF7	Inhibitor of CLKs	SM08502 inhibits SRSF phosphorylation and disrupts spliceosome activity, which inhibits of Wnt pathway-related gene and expression of splicing regulator.	Preclinical Cell level Mice level Phase 1 clinical trial (NCT03355066)	[116]
SSO(Dex8-VDR oligomer SA (+12))	VDR	SSO target splice acceptor site of exon8	SSO targets splice acceptor site of exon8 and alters VDR signaling cascades for treatment.	Preclinical Cell level	[109]
Nicotinamide(NAM):(HDAC I and II inhibitors)	KDM3A	Block the kinase activity of HDAC to decrease PHF5A acetylation	The acetylated PHF5A interacts with U2 snRNP complex and reduces aberrant splicing of KDM3A and the inhibitor blocks PHF5A acetylation.	Preclinical Cell level	[70]
FR901464(Small molecular inhibitor)	–	FR901464 competitively binds to SF3B1	The inhibitor binds with and inhibits SF3B1 and destabilizes the recruitment of snRNP U2 and spliceosome assembly to decreases cell proliferation and tumor growth.	Preclinical	[114]
Pladienolide B	SF3B1	Inhibitor of SF3B1 assembly	The inhibitor promotes cell apoptosis by the alternative use of two 5’ SS regions in exon 2 and increases BCL-xs isoform.	Preclinical	[80, 81]
E7107	SF3B1	Inhibitor of SF3B1 assembly	The inhibitor reduced remodeling U2 snRNP to expose the branch point-binding region and then inhibits tumor growth.	Phase 1 clinical trial	[138]
Indacaterol (SRSF6 inhibitor)	ZO-1	Binding to RRM2 domain of SRSF6	The inhibitor can reduce RRM2 binding to ZO-1 exon23 and suppress CRC tumourigenicity.	Preclinical Cell level Mice level	[59]

*SSO* Splice-Switching Oligonucleotides, *3’SS* 3’ splice site, *5’ SS* 5’ splice site.

### Therapeutic approaches of antisense oligonucleotides

A more direct method of regulating a specific splicing event is by exploring splice-switching oligonucleotides (SSOs), which belong to ASOs with splicing intervention functions. Briefly, SSOs typically consist of sequences of ~15–30 nucleotides that are chemically modified to avoid degradation by exonucleases, and bind to a unique sequence on the mRNA [107]. Intravenous and subcutaneous injection were supposed to be the high-efficiency delivery strategies at the present period. Some modifications of the SSOs like 2ʹ-O-methyl (2ʹ-OMe) and 2ʹ-O-methoxy-ethyl (2ʹ-MOE) help to maintain the concentration by avoiding degradation by nuclease in blood [108]. These modifications help SSOs to avoid lysosomal degradation and enter the cytoplasm or nucleus to execute their pharmacological functions. For instance, PMO of Dex8 VDR_helps to remain stable in CACO2 cell cytoplasm [109]. SSOs uptake, which required binding to surface proteins, increased a lot in target cells if packaged with lipid nanoparticle delivery system [110].

Generally, SSOs can be specifically designed into: (1) those targeting direct splicing sites, including 5’SS, 3’SS or branch site thus blocking their usage, (2) those targeting cis-regulation elements of splicing by intervening recognition of splicing factor, such as splicing enhancer sequences or splicing silencer sequences [109, 111]. In colon cancer cells, two SSOs targeting BCL-2-associated transcription factor 1 (BCLAF1) pre-mRNA have been demonstrated to effectively inhibit cell growth through splicing regulation. High-expression SRSF10 in tumor cells regulates the inclusion of exon5a of BCLAF1 and increases the generation of pro-growth full-length variants with exon 5a inclusion. The two SSOs, targeting 3’SS at the boundary of intron4 and exon5a of BCLAF1, can suppress tumor cell proliferation by reducing full-length variants under the regulation of SRSF10 [61]. A previous study revealed that SSO targeting 3′SS of intron 2 of DVL2 enhances intron 2 retention variant in DVL2, which inhibits CRC cell proliferation in vitro [46]. Furthermore, SSOs targeting VEGFR or SMN2 splicing site inhibited tumor growth by reducing oncogenic variants in CRC (See Fig. 4A and Table 3).

However, systemic use of SSOs for the treatment of solid tumors remains a challenge due to limited drug distribution, similar to the use of SSOs to treat muscular diseases [112]. Thus, SSOs may be an effective treatment agent without off-target effects and high specificity if the aforementioned challenges are addressed. RNA-based therapeutics offer the potential to virtually target molecules especially those lacking catalytic activities that could be inhibited, or molecules that are not responsive to targeted antibody approaches [113].

### Therapeutic approaches of small molecule inhibitors

Small molecule drugs that exhibit potent anticancer activity have been developed by targeting splicing factors to modulate their activities or products that promote tumor growth by splicing. Nevertheless, small molecule drugs could potentially inhibit splicing but they lack specificity when compared with SSOs therapy. Small molecule inhibitors targeting AS are listed in Fig. 4B, C and Table 3.

#### Targeting of core spliceosome complex and regulatory proteins

Application of indacaterol, which is an inhibitor that targets the functional RRM2 domain of SRSF6, can inhibit SRSF6 and binding to exon23 of tight junction protein 1 (ZO-1) in splicing regulation, which effectively reduces CRC progression [59]. SF3B1 is a crucial spliceosome component that participates in the splicing and synthesis of mature mRNA. FR901464 is a small molecule inhibitor that binds to SF3B1 and can destabilize the recruitment of snRNP U2 to SF3B1, which ultimately decreases cell proliferation and tumor growth [114]. Moreover, certain molecules that target splicing regulation factors exhibit anti-cancer activity in CRC. For example, 10058-F4 (MYC inhibitor) suppresses the expression of epithelial splicing regulatory protein 2 (ESRP2) and SM08502 (CLKs inhibitor) decreases phosphorylation of SRSFs [115, 116].

#### Targeting of splicing variants or translation products or downstream targets

Some drugs have been developed to specifically target splicing variants or their translation isoforms to inhibit tumor progression, like Prodigiosin (targeting ΔNp73) [117], Nilotinib (targeting ZAK) [118], SB21673 (targeting ITGA6A) [115], etc. (Refer to Table 3 for detail).

The *BTK* gene can be developed as a multi-target agent because of its complex splicing mechanisms. P65BTK is an oncogenic isoform and is dependent on splicing by p-hnRNPK, which is phosphorylated by signal-regulated protein kinases-1/2 (ERK1/2) and RAS. Not only Ibrutinib and AVL-292 (inhibitors of P65BTK), but also the FTI-277 (inhibitors of p-hnRNPK, targeting RAS) and CI-1040 (inhibitors of p-hnRNPK, targeting MEK1/2), can suppress tumor progression [119]. The multi-target agents could provide novel strategies that address drug resistance and enhance therapeutic effects.

Notably, certain inhibitors targeting downstream of specific splicing products can also be used as potential treatment strategies. Thioredoxin-like protein 2 variant 2 (Txl-2b), a specific isoform upregulated in CRC by splicing, activates NF-κB signaling and induces NF-κB-regulated gene products, but other isoforms do not perform such functions. Treatment with Bay 11-7082 (NF-κB inhibitor) enhances the sensitivity of tumor cells against vincristine and induces apoptosis in vitro, which shows the potential of combining treatment in drug-resistance CRC [120].

### Others therapeutic targets

Specific aberrant RNA sequences or structures (such as hairpins or G-quadruplexes) can block recruitment of splicing factors to splicing sequences, which could serve as novel therapeutic targets for CRC. Two G4s are detected at 118 bp upstream and 261 bp downstream of the acceptor sites of the exons 4 and 7 of CD133, respectively. As an exogenous mimic of G sequence, RHPS4 targets G-quadruplexes (G4s) leading to a marked reduction of CD133 mature transcripts that is counterbalanced by an increase in splicing variants corresponding to enhanced intron retention, which ultimately inhibits tumor progression [121].

## Conclusion

RNA splicing is a molecular event that occurs after the posttranscriptional gene expression processes in invertebrates. Aberrant splicing is associated with the development of diseases, such as cancer. Advances in nucleic acid sequencing and computational biology increase our understanding of the relationship between colorectal cancer and AS [64, 122]. The emergence of single-cell sequencing technology makes it possible to screen out one or several splice variants in tumor cells that are not present in heterogeneous tumor tissues [123]. In addition, it is emerging from previous studies that non-coding RNA splicing has important pathological implications. For example, circular RNAs are by-products of back-splicing of pre-mRNA and regulate biological processes by acting either as sponges of microRNAs (miRNAs) or RNA-binding proteins. Research on the roles of circRNA in CRC is still in its infancy stage and hence further studies are advocated to expose the potential of using circRNA as biomarkers and therapeutic targets [124].

In this review, we summarized findings from research on the role of AS in CRC in the past 5 years and reveal their clinical value. Inaccurate sequences on cis-elements and changes in the trans-regulation factors result in aberrant AS or new splicing events. This affects malignant hallmarks such as proliferation, apoptosis, invasion, migration, and drug resistance. AS signature profiles or patterns can be used as disease biomarkers. Some specific splicing variants are associated with tumor grade and prognosis of patients, and can be applied in clinical practice. For instance, KRAS-A and VEGFA 165b variant are superior predictors of overall survival whereas CD44v6, CEACAM1-Short isoform, and Δ133p53β variant are poor predictors of overall survival among patients with CRC [59, 85, 88, 90, 97, 98, 104–106]. SSOs and small molecular drugs that target various aspects of splicing progress or products can suppress tumor progression, some of which have been investigated in phase I clinical trials, such as E7107 and SM08502.

However, the function of AS in the progression of CRC is still poorly understood, at least compared to prostatic cancer or breast cancer. Even so, they offer hope with exposing novel mechanism of low toxicity and new opportunity for the use of SSOs in precision medicine with less off-target effect. Although SSOs confer some benefits in patients with Duchenne muscular dystrophy (DMD) [125], its role in CRC patients has not been sufficiently explored [126]. It is worth mentioning that the high polarity and charged characteristics of oligonucleotide drugs make them obvious differences between small chemical molecules and monoclonal antibody drugs in terms of drug delivery system, pharmacokinetic properties, and efficacy [110]. Although significant progress has been made, the tumor-specific splicing alterations are not fully characterized and further efforts are needed to comprehensively reveal the regulation splicing of AS events.

## Supplementary information

supplementary table
