# Supplementary material for: Alternative splicing of mRNA in colorectal cancer: new strategies for tumor diagnosis and treatment
Source: Cell Death Dis. 2021 Jul 30;12(8):752. doi: 10.1038/s41419-021-04031-w (PMC8324868; doi:10.1038/s41419-021-04031-w)
Supplement: Supplementary file 1 — supplementary table [file 41419_2021_4031_MOESM1_ESM.docx]

**Supplementary table 1: Alternative splicing products and their function in colorectal cancer.**

| **Hallmarks** | **Gene** | **Splicing event** | **Transcript variant** | **Biological function** | **Splicing factor** | **Experimental evidence** | **Referrence** | **Clinical Application** |
| --- | --- | --- | --- | --- | --- | --- | --- | --- |
| Proliferation neomycin- resistance | BCLAF1 | Exon 5 skipping | BCLAF1 full length | Pro proliferation Pro neomycin resistance | SRSF10 | Cell line Xenograft | ^1^ | Correlated with higher tumor grade. |
|  |  |  | BCLAF1 truncated | Anti proliferation Anti neomycin resistance |  |  |  |  |
| Proliferation | BTK | Exon 1A skipping | P65BTK | Pro proliferation | hnRNPK | Cell line | ^2^ | High expression in tumor than adjacent tissue. |
|  |  |  | P77BTK | Anti proliferation |  |  |  |  |
| Proliferation | CD133 | Intron inclusion | CD133 intron 3 inclusion | Anti proliferation | - | Cell line Xenograft | ^3^ | - |
|  |  |  | CD133 intron 6 inclusion | Anti proliferation |  |  |  | - |
| Proliferation stemness invasion | CD44 | Contains v4-v10 exons | CD44 v4-10 | Pro proliferation | - | Cell line Organoid Xenograft Primary model Xenopus laevis | ^4-6^ | - |
|  |  |  | CD44 standard | Anti proliferation |  |  |  | - |
| Proliferation | CIZ1 | Exon 8-12 skipping | CIZ1-F | Pro proliferation | - | Cell line | ^7^ | - |
|  |  |  | CIZ full-length | - |  |  |  | - |
| Proliferation | CK1 | - | CK1δ | - | - | Cell line Xenograft | ^8^ | - |
|  |  |  | CK1ε | - |  |  |  | Marker of better survival |
| Proliferation invasion migration | CXCR3 | - | CXCR3A | Pro proliferation Anti invasion and migration | - | Cell line | ^9^ | TNM marker I,II/III,IV |
|  |  |  | CXCR3B | Anti proliferation Pro invasion and migration |  | Xenograft | ^10^ | - |
| Proliferation | DBF4B | Exon 6 skipping | DBF4B full-length | Pro proliferation | SRSF1 | Cell line | ^11^ | BF4BFL/S ratio is negative marker of survival |
|  |  |  | DBF4B-s | Anti proliferation |  | Xenograft |  | - |
| EMT | Rac1 | Inclusion of exon 3b | RAC1 isoform b | Pro proliferation | PTBP1 | Data analysis | ^12^ | - |
|  |  |  | Rac1 isoform a | Anti proliferation |  |  |  | - |
| Proliferation | NUMB | Exon 9 inclusion | NUMB Isoform 1 | Pro proliferation | PTBP1 | Data analysis | ^12^ | - |
|  |  |  | NUMB Isoform 2 | Anti proliferation |  |  |  | - |
| Proliferation apoptosis | Fas | Exon 6 skipping | Fas-short | Pro proliferation Anti apoptosis | SRSF7 | Cell line Data analysis | ^13, 14^ | - |
|  |  |  | Fas-long | Anti proliferation Pro apoptosis |  |  |  | - |
| Proliferation | GPR137 | Exon 2-3 skipping | GPR137-long | Pro proliferation | ESRP1 | Cell line | ^15^ | low ratio of GPR137_Short / Long is negative survival |
|  |  |  | GPR137-short | Anti proliferation |  |  |  | - |
| Proliferation | HIF-3α | - | HIF-3α1(long isoform) | Pro proliferation | - | Cell line | ^16^ | - |
|  |  |  | HIF-3α2 | - |  |  |  | - |
| Proliferation differation | HNF4a | Exon1 skipping | P1-HNF4a isoform(HNF4a1,2) | Anti proliferation Pro differation | - | Cell line Organoid Xenograft Primary model Data analysis | ^17-19^ | - |
|  |  |  | P2-HNF4a(HNF4a7,8) | Pro proliferation Anti differation |  |  |  | - |
| Proliferation | ITGA6 | Exon25 skipping | ITGA6A | Pro proliferation | ESRP2 | Cell line Data analysis | ^20, 21^ | - |
|  |  |  | ITGA6B | - |  |  |  | - |
| Proliferation apoptosis | PKM | Exon skipping | PKM1 | Anti proliferation Pro apoptosis | hnRNPA1 | Cell line Xenograft Data analysis | ^22-26^ | - |
|  |  |  | PKM2 | Pro proliferation Anti apoptosis | SRSF3 |  |  | - |
| Proliferation | TIF‐IA | - | TIF‐90 | Pro proliferation | - | Cell line | ^27^ | - |
|  |  |  | TIF-FL | Anti proliferation |  |  |  | - |
| Proliferation | TRA2β | Exon 2 inclusion | TRA2β4(with exon 2) | Pro proliferation Anti apoptosis | - | Cell line | ^28, 29^ | - |
| Apoptosis |  |  | TRA2β1 | Anti proliferation Pro apoptosis |  |  |  | - |
| Proliferation | HNRPLL | Exon 12 inclusion | HNRPLL E12A RNA | Pro proliferation | - | Cell line | ^30^ | - |
|  |  |  |  |  |  |  |  |  |
|  |  |  | HNRPLL | Anti proliferation |  |  |  | - |
| Proliferation | ID1 | - | ID1a | Pro proliferation Anti metastasis | - | Cell line | ^31^ | - |
| Metastasis |  |  | ID1b | Anti proliferation Pro metastasis |  | Xenograft |  | - |
| Proliferation apoptosis | KLF6 | Exon2 skipping | KLF6-SV2 | Anti proliferation Pro apoptosis | - | Cell line | ^32^ | No significance with survival or grade. |
|  |  |  | KLF6-SV1,3 | - |  |  |  |  |
| Proliferation  invasion  metastasis | ZO-1 | Exon23 skipping | ZO-1 exon23 skipping isoform | Pro proliferation Anti invasion and metastasis | SRSF6 | Cell line  Xenograft  Primary model | ^33^ | Splicing ratio is negative survival marker. |
|  |  |  | ZO-1 exon23 inclusion isoform | Anti proliferation Pro invasion and metastasis |  |  |  | - |
| Proliferation | ZAK | exon 12 inclusion | ZAK-LF | Pro proliferation | PRPF6 | Cell line  Xenograft  Data analysis | ^34^ | - |
|  |  |  | ZAK-SF | - |  |  |  | - |
| Proliferation | P73 | - | N-terminally truncated isoform ΔNp73 | Pro proliferation |  | Cell line Xenograft | ^35^ | - |
|  |  |  | P73 full-length | - |  |  |  | - |
| Proliferation apoptosis | MNK2 | exon 14a skipping | MNK2a | Anti proliferation Pro apoptosis | SRSF1 | Cell line | ^36^ | - |
|  |  |  | MNK2b(lack) | Pro proliferation Anti apoptosis |  |  |  | - |
| Proliferation | Tcf1 | - | Tcf1-long | Pro proliferation | - | Cell line Organoid Data analysis | ^37^ | - |
|  |  |  | Tcf1-short | - |  |  |  | - |
| Proliferation apoptosis | MRPL33 | exon 3 inclusion | MRPL33-L | Pro proliferation Anti apoptosis | hnRNPK | Cell line | ^38^ | - |
|  |  |  | MRPL33-S | Anti proliferation Pro apoptosis |  |  |  | - |
| Proliferation apoptosis vincristine resistance | Txl-2 | exon skipping | Txl-2b (splice variant lacking exons 1 and 5) | Pro proliferation Anti apoptosis Pro vincristine resistance | - | Cell line Xenograft | ^39^ | - |
|  |  |  | full length | - |  |  |  | - |
|  |  |  | Txl-2 (Txl-2a) |  |  |  |  |  |
|  |  |  | Txl-2c (splice variant lacking exons 1, 4 and 5) |  |  |  |  |  |
| 5-FU resistance | LGR5 | exon skipping | LGR5 full-length | Pro 5-FU Resistance | - | Cell line | ^40^ | High ratio of LGR5-FL refer to chemotherapy resistance. |
|  |  |  | LGR5Δ5 | Anti 5-FU Resistance |  |  |  | - |
|  |  |  | LGR5Δ5-8 |  |  |  |  |  |
| Angiogenesis proliferation bevacizumab Resistance | TIA-1 | exon 6a skipping | TIA-1s | Pro angiogenesis Pro proliferation Pro bevacizumab Resistance | - | Cell line Xenograft | ^41^ | - |
|  |  |  | TIA-1 full-length | Anti angiogenesis Anti proliferation Anti bevacizumab Resistance |  |  |  | - |
| Proliferation | PADI2 | intron11 inclusion | PADI2Isoform1 | Pro proliferation | - | Cell line | ^42^ | - |
|  |  |  | PADI2Isoform2 | Anti proliferation |  |  |  | - |
| Proliferation | STAT3 | - | STAT3-α　(full- length) | Pro proliferation | - | Cell line Xenograft | ^43^ | - |
|  |  |  | STAT3-β(truncated) | - |  |  |  | - |
| Proliferation stemness | OCT4 | - | OCT4B1 | Pro proliferation Pro stemness | - | Cell line | ^44^ | - |
|  |  |  | OCT4B2 | - |  |  |  | - |
| Proliferation apoptosis | WNT-5a | alternative promoters A and B | Wnt-5a short | Pro proliferation Anti apoptosis | - | Cell line | ^45^ | High Wnt5a-S and low Wnt5a-L mRNA were significantly positively correlated with tumor depth. |
|  |  |  | Wnt-5a long | Anti proliferation Pro apoptosis |  |  |  |  |
| Apoptosis bleomycin resistance | RAF1 | exon 11 inclusion | Raf1-tr | Pro apoptosis Anti bleomycin resistance | - | Cell line Data analysis | ^46^ | - |
|  |  |  | Raf1-full length | Anti apoptosis Pro bleomycin resistance |  |  |  | - |
| Apoptosis | Bax | exon2 skipping | Bax∆2 | Pro apoptosis | - | Cell line | ^47^，^48^ | - |
|  |  |  | Baxα | Pro apoptosis |  |  |  | - |
| Invasion apoptosis | BARD1 | - | BARD1β | Pro invasion Anti apoptosis | - | Cell line | ^49^ | - |
|  |  |  | BARD1-fl | Anti invasion Pro apoptosis |  |  |  | - |
| Proliferation metastasis | AURORA | exon2 contain | AURORA-A | Pro proliferation Pro metastasis | - | CCell line Xenograft | ^50^ | - |
|  |  |  | - | - |  |  |  | - |
| Invasion 5-FU resistance | CD44 | exon variant 6 inclusion | CD44 v6 | Pro invasion Pro 5-FU Resistance | Hnrnpll | Cell line Organoid Primary model Xenograft Data analysis | ^51-55^ | CD44v6 is a useful marker for predicting patient with poor prognosis. |
|  |  |  | CD44 S | - |  |  |  |  |
| Invasion | CD44 | Exon variant 9 inclusion | CD44 v9 | Anti invasion | ESRP1 | Cell line | ^56^ | High ratio of CD44v9/CD44s is a good marker of DFS. |
|  |  |  | CD44 S | Pro invasion |  |  |  |  |
| Invasion metastasis | CEACAM1 | Exon 7 skipping | CEACAM1-L | Anti invasion and metastasis | HnRNPL | Cell line Xenograft | ^57-60^ | - |
|  |  |  | CEACAM1-S | Pro invasion and metastasis |  |  |  | CEACAM1-S is a marker of poor OS and DFS. |
| Invasion metastasis | NRP1 | Exon skipping | NRP1-ΔE4 | Pro invasion and metastasis | - | Cell line Xenograft | ^61^ | Stage I–IV patients showed that NRP1-ΔE4 was positively expressed in higher tumor grade. |
|  |  |  | NRP1-ΔE5 |  |  |  |  | - |
|  |  |  | NRP1 full-length | NS |  |  |  | - |
| Metastasis | FAK | Exons 13, 14, 16, | FAK0 | - | - | Cell line Xenograft | ^62^ | - |
|  |  | and 31 | FAK6 | Pro metastasis |  |  |  | FAK6 and FAK28 are better treatment targets. |
|  |  |  | FAK28 | Pro metastasis |  |  |  |  |
| Metastasis | MAP4K | Exon 16,17 skipping | MAP4K isoform 2 | Pro metastasis | SRSF3 | Cell line | ^63^ | - |
|  |  |  | MAP4K isoform 5 | Pro metastasis |  |  |  |  |
|  |  |  | MAP4K isoform 1 | Anti metastasis |  |  |  | - |
| Invasion migration | ZAK | - | ZAK-α | Pro invasion and migration | - | Cell line | ^64^ | - |
|  |  |  | ZAK-β | Pro invasion and migration |  |  |  | - |
| Metastasis | P53 | Intron-4 inclusion | Δ133p53β | Pro metastasis | - | Cell line Xenograft | ^65^ | Δ133p53β positive is a marker of poor survival. |
|  |  |  | Δ133p53γ | - |  |  |  | - |
|  |  |  | Δ133p53α | - |  |  |  |  |
| Angiogenesis  Bevacizumab Resistance | VEGF | Skipping of exon 6a,7a,8a | VEGFA 165b (without exon8a) | Anti angiogenesis Anti Bevacizumab Resistance | TIA-1 SRSF6 | Cell line Primary Model Xenograft Data analysis | ^66^ | VEGFA 165b is a favoural prognosis of CRC. |
|  |  |  |  |  |  |  | ^41, 67^ |  |
|  |  |  | VEGFA 165a | Pro angiogenesis | - |  | ^67-70^ |  |
|  |  |  | VEGFA 121a | Pro angiogenesis |  |  | ^67, 70^ |  |
|  |  |  | VEGFA 121b | Pro angiogenesis |  |  |  |  |
|  |  |  | VEGFA 189a | Pro angiogenesis |  |  | ^71^ |  |
|  |  |  | VEGF 145a | Pro angiogenesis |  |  | ^67^ |  |
|  |  |  | VEGF 145b | Anti angiogenesis |  |  | ^67^ |  |
| Angiogenesis | VEGFR2 | Intron13 inclusion | mVEGFR2 | Pro angiogenesis | - | Cell line Xenograft | ^72^ | - |
|  |  |  | sVEGFR2 | Anti angiogenesis |  |  |  | - |
| Proteasome inhibitors Resistance( bortezomib and carfilzomib) | BAX | Exon2,3 skipping | Bax∆2 | Anti proteasome inhibitors resistance | - | Cell line Xenograft | ^47, 48^ | - |
|  |  |  | Bax full-length | Anti proteasome inhibitors resistance |  |  |  | - |
| 5-FU and cisplatin resistance | LIN28B | Exon1 skipping | LIN28B-long | Pro 5-FU and cisplatin resistance | - | Cell line Xenograft Organoid | ^73^ | - |
|  |  |  | LIN28B-short | Anti 5-FU and cisplatin resistance |  |  |  | - |
| Apoptosis | BCL2L1 | Alternative 5’SS in exon 2 | BCL-xl isoform | Anti apoptosis | hnRNPA2B1 | Data analysis  Cell line | ^74, 75^ | - |
|  |  |  | BCL-xs isoform | Pro apoptosis |  |  |  |  |
| Apoptosis | KRAS | Mutually exclusive exon 4A or 4B | KRAS 4A | Pro apoptosis | RBM38 |  | ^76^ | KRAS4A expression is associated with a better overall survival. |
|  |  |  | KRAS 4B | Anti apoptosis |  |  |  |  |

1. Zhou X, Li X, Cheng Y, Wu W, Xie Z, Xi Q*, et al.* BCLAF1 and its splicing regulator SRSF10 regulate the tumorigenic potential of colon cancer cells. *Nat Commun* **5**, 4581(2014).

2. Grassilli E, Pisano F, Cialdella A, Bonomo S, Missaglia C, Cerrito MG*, et al.* A novel oncogenic BTK isoform is overexpressed in colon cancers and required for RAS-mediated transformation. *Oncogene* **35**, 4368-4378(2016).

3. Zizza P, Cingolani C, Artuso S, Salvati E, Rizzo A, D'Angelo C*, et al.* Intragenic G-quadruplex structure formed in the human CD133 and its biological and translational relevance. *Nucleic Acids Res* **44**, 1579-1590(2016).

4. Joosten SPJ, Zeilstra J, van Andel H, Mijnals RC, Zaunbrecher J, Duivenvoorden AAM*, et al.* MET Signaling Mediates Intestinal Crypt-Villus Development, Regeneration, and Adenoma Formation and Is Promoted by Stem Cell CD44 Isoforms. *Gastroenterology* **153**, 1040-1053 e1044(2017).

5. Zeilstra J, Joosten SP, van Andel H, Tolg C, Berns A, Snoek M*, et al.* Stem cell CD44v isoforms promote intestinal cancer formation in Apc(min) mice downstream of Wnt signaling. *Oncogene* **33**, 665-670(2014).

6. Schmitt M, Metzger M, Gradl D, Davidson G, Orian-Rousseau V. CD44 functions in Wnt signaling by regulating LRP6 localization and activation. *Cell Death Differ* **22**, 677-689(2015).

7. Swarts DRA, Stewart ER, Higgins GS, Coverley D. CIZ1-F, an alternatively spliced variant of the DNA replication protein CIZ1 with distinct expression and localisation, is overrepresented in early stage common solid tumours. *Cell Cycle* **17**, 2268-2283(2018).

8. Richter J, Ullah K, Xu P, Alscher V, Blatz A, Peifer C*, et al.* Effects of altered expression and activity levels of CK1delta and varepsilon on tumor growth and survival of colorectal cancer patients. *Int J Cancer* **136**, 2799-2810(2015).

9. Li H, Rong S, Chen C, Fan Y, Chen T, Wang Y*, et al.* Disparate roles of CXCR3A and CXCR3B in regulating progressive properties of colorectal cancer cells. *Mol Carcinog* **58**, 171-184(2019).

10. Jin J, Zhang Z, Wang H, Zhan Y, Li G, Yang H*, et al.* CXCR3 expression in colorectal cancer cells enhanced invasion through preventing CXCR4 internalization. *Exp Cell Res* **371**, 162-174(2018).

11. Chen L, Luo C, Shen L, Liu Y, Wang Q, Zhang C*, et al.* SRSF1 Prevents DNA Damage and Promotes Tumorigenesis through Regulation of DBF4B Pre-mRNA Splicing. *Cell Rep* **21**, 3406-3413(2017).

12. Hollander D, Donyo M, Atias N, Mekahel K, Melamed Z, Yannai S*, et al.* A network-based analysis of colon cancer splicing changes reveals a tumorigenesis-favoring regulatory pathway emanating from ELK1. *Genome Res* **26**, 541-553(2016).

13. Fu Y, Wang Y. SRSF7 knockdown promotes apoptosis of colon and lung cancer cells. *Oncol Lett* **15**, 5545-5552(2018).

14. Jakubauskiene E, Peciuliene I, Vilys L, Mocevicius P, Vilkaitis G, Kanopka A. Gastrointestinal tract tumors and cell lines possess differential splicing factor expression and tumor associated mRNA isoform formation profiles. *Cancer Biomark* **15**, 575-581(2015).

15. Mager LF, Koelzer VH, Stuber R, Thoo L, Keller I, Koeck I*, et al.* The ESRP1-GPR137 axis contributes to intestinal pathogenesis. *Elife* **6**, (2017).

16. Xue X, Jungles K, Onder G, Samhoun J, Gyorffy B, Hardiman KM. HIF-3alpha1 promotes colorectal tumor cell growth by activation of JAK-STAT3 signaling. *Oncotarget* **7**, 11567-11579(2016).

17. Chellappa K, Deol P, Evans JR, Vuong LM, Chen G, Briancon N*, et al.* Opposing roles of nuclear receptor HNF4alpha isoforms in colitis and colitis-associated colon cancer. *Elife* **5**, (2016).

18. Babeu JP, Jones C, Geha S, Carrier JC, Boudreau F. P1 promoter-driven HNF4alpha isoforms are specifically repressed by beta-catenin signaling in colorectal cancer cells. *J Cell Sci* **131**, (2018).

19. Vuong LM, Chellappa K, Dhahbi JM, Deans JR, Fang B, Bolotin E*, et al.* Differential Effects of Hepatocyte Nuclear Factor 4alpha Isoforms on Tumor Growth and T-Cell Factor 4/AP-1 Interactions in Human Colorectal Cancer Cells. *Mol Cell Biol* **35**, 3471-3490(2015).

20. Groulx JF, Boudjadi S, Beaulieu JF. MYC Regulates alpha6 Integrin Subunit Expression and Splicing Under Its Pro-Proliferative ITGA6A Form in Colorectal Cancer Cells. *Cancers (Basel)* **10**, (2018).

21. Groulx JF, Giroux V, Beausejour M, Boudjadi S, Basora N, Carrier JC*, et al.* Integrin alpha6A splice variant regulates proliferation and the Wnt/beta-catenin pathway in human colorectal cancer cells. *Carcinogenesis* **35**, 1217-1227(2014).

22. Kuranaga Y, Sugito N, Shinohara H, Tsujino T, Taniguchi K, Komura K*, et al.* SRSF3, a Splicer of the PKM Gene, Regulates Cell Growth and Maintenance of Cancer-Specific Energy Metabolism in Colon Cancer Cells. *Int J Mol Sci* **19**, (2018).

23. Huang JZ, Chen M, Chen, Gao XC, Zhu S, Huang H*, et al.* A Peptide Encoded by a Putative lncRNA HOXB-AS3 Suppresses Colon Cancer Growth. *Mol Cell* **68**, 171-184 e176(2017).

24. Sun Y, Luo M, Chang G, Ren W, Wu K, Li X*, et al.* Phosphorylation of Ser6 in hnRNPA1 by S6K2 regulates glucose metabolism and cell growth in colorectal cancer. *Oncol Lett* **14**, 7323-7331(2017).

25. Liang F, Li Q, Li X, Li Z, Gong Z, Deng H*, et al.* TSC22D2 interacts with PKM2 and inhibits cell growth in colorectal cancer. *Int J Oncol* **49**, 1046-1056(2016).

26. Taniguchi K, Sugito N, Kumazaki M, Shinohara H, Yamada N, Nakagawa Y*, et al.* MicroRNA-124 inhibits cancer cell growth through PTB1/PKM1/PKM2 feedback cascade in colorectal cancer. *Cancer Lett* **363**, 17-27(2015).

27. Nguyen DQ, Hoang DH, Nguyen TTV, Ho HD, Huynh V, Shin JH*, et al.* Ebp1 p48 promotes oncogenic activities in human colon cancer cells through regulation of TIF-90-mediated ribosomal RNA synthesis. *J Cell Physiol* **234**, 17612-17621(2019).

28. Satake Y, Kuwano Y, Nishikawa T, Fujita K, Saijo S, Itai M*, et al.* Nucleolin facilitates nuclear retention of an ultraconserved region containing TRA2beta4 and accelerates colon cancer cell growth. *Oncotarget* **9**, 26817-26833(2018).

29. Kajita K, Kuwano Y, Satake Y, Kano S, Kurokawa K, Akaike Y*, et al.* Ultraconserved region-containing Transformer 2beta4 controls senescence of colon cancer cells. *Oncogenesis* **5**, e213(2016).

30. Chen YT, Chang IY, Liu H, Ma CP, Kuo YP, Shih CT*, et al.* Tumor-associated intronic editing of HNRPLL generates a novel splicing variant linked to cell proliferation. *J Biol Chem* **293**, 10158-10171(2018).

31. Manrique I, Nguewa P, Bleau AM, Nistal-Villan E, Lopez I, Villalba M*, et al.* The inhibitor of differentiation isoform Id1b, generated by alternative splicing, maintains cell quiescence and confers self-renewal and cancer stem cell-like properties. *Cancer Lett* **356**, 899-909(2015).

32. Zhang B, Guo DD, Zheng JY, Wu YA. Expression of KLF6-SV2 in colorectal cancer and its impact on proliferation and apoptosis. *Eur J Cancer Prev* **27**, 20-26(2018).

33. Wan L, Yu W, Shen E, Sun W, Liu Y, Kong J*, et al.* SRSF6-regulated alternative splicing that promotes tumour progression offers a therapy target for colorectal cancer. *Gut* **68**, 118-129(2019).

34. Adler AS, McCleland ML, Yee S, Yaylaoglu M, Hussain S, Cosino E*, et al.* An integrative analysis of colon cancer identifies an essential function for PRPF6 in tumor growth. *Genes Dev* **28**, 1068-1084(2014).

35. Prabhu VV, Hong B, Allen JE, Zhang S, Lulla AR, Dicker DT*, et al.* Small-Molecule Prodigiosin Restores p53 Tumor Suppressor Activity in Chemoresistant Colorectal Cancer Stem Cells via c-Jun-Mediated DeltaNp73 Inhibition and p73 Activation. *Cancer Res* **76**, 1989-1999(2016).

36. Maimon A, Mogilevsky M, Shilo A, Golan-Gerstl R, Obiedat A, Ben-Hur V*, et al.* Mnk2 Alternative Splicing Modulates the p38-MAPK Pathway and Impacts Ras-Induced Transformation. *Cell Reports* **7**, 501-513(2014).

37. Shiokawa D, Sato A, Ohata H, Mutoh M, Sekine S, Kato M*, et al.* The Induction of Selected Wnt Target Genes by Tcf1 Mediates Generation of Tumorigenic Colon Stem Cells. *Cell Rep* **19**, 981-994(2017).

38. Liu L, Luo C, Luo Y, Chen L, Liu Y, Wang Y*, et al.* MRPL33 and its splicing regulator hnRNPK are required for mitochondria function and implicated in tumor progression. *Oncogene* **37**, 86-94(2018).

39. Lu Y, Zhao X, Luo G, Shen G, Li K, Ren G*, et al.* Thioredoxin-like protein 2b facilitates colon cancer cell proliferation and inhibits apoptosis via NF-kappaB pathway. *Cancer Lett* **363**, 119-126(2015).

40. Osawa H, Takahashi H, Nishimura J, Ohta K, Haraguchi N, Hata T*, et al.* Full-length LGR5-positive cells have chemoresistant characteristics in colorectal cancer. *Br J Cancer* **114**, 1251-1260(2016).

41. Hamdollah Zadeh MA, Amin EM, Hoareau-Aveilla C, Domingo E, Symonds KE, Ye X*, et al.* Alternative splicing of TIA-1 in human colon cancer regulates VEGF isoform expression, angiogenesis, tumour growth and bevacizumab resistance. *Molecular Oncology* **9**, 167-178(2015).

42. Funayama R, Taniguchi H, Mizuma M, Fujishima F, Kobayashi M, Ohnuma S*, et al.* Protein-arginine deiminase 2 suppresses proliferation of colon cancer cells through protein citrullination. *Cancer Sci* **108**, 713-718(2017).

43. Marino F, Orecchia, V., Regis, G., Musteanu, M., Tassone, B., Jon, C., Forni, M., Calautti, E., Chiarle, R., Eferl, R., & Poli, V. . STAT3β controls inflammatory responses and early tumor onset in skin and colon experimental cancer models. . *American journal of cancer research* **4**, 484–494(2014).

44. Wen KM, Zhang GH, Li J, Chen ZQ, Cheng YL, Su X*, et al.* OCT4B1 promotes cell growth, migration and invasion suppressing sensitivity to omicronxaliplatin in colon cancer. *Oncol Rep* **34**, 2943-2952(2015).

45. Huang TC, Lee PT, Wu MH, Huang CC, Ko CY, Lee YC*, et al.* Distinct roles and differential expression levels of Wnt5a mRNA isoforms in colorectal cancer cells. *PLoS One* **12**, e0181034(2017).

46. Nixon BR, Sebag SC, Glennon MS, Hall EJ, Kounlavong ES, Freeman ML*, et al.* Nuclear localized Raf1 isoform alters DNA-dependent protein kinase activity and the DNA damage response. *FASEB J* **33**, 1138-1150(2019).

47. Zhang H, Lin Y, Manas A, Zhao Y, Denning MF, Ma L*, et al.* BaxDelta2 promotes apoptosis through caspase-8 activation in microsatellite-unstable colon cancer. *Mol Cancer Res* **12**, 1225-1232(2014).

48. Manas A, Chen W, Nelson A, Yao Q, Xiang J. BaxDelta2 sensitizes colorectal cancer cells to proteasome inhibitor-induced cell death. *Biochem Biophys Res Commun* **496**, 18-24(2018).

49. Ozden O, Bishehsari F, Bauer J, Park SH, Jana A, Baik SH*, et al.* Expression of an Oncogenic BARD1 Splice Variant Impairs Homologous Recombination and Predicts Response to PARP-1 Inhibitor Therapy in Colon Cancer. *Sci Rep* **6**, 26273(2016).

50. Lai CH, Chen RY, Hsieh HP, Tsai SJ, Chang KC, Yen CJ*, et al.* A selective Aurora-A 5'-UTR siRNA inhibits tumor growth and metastasis. *Cancer Lett* **472**, 97-107(2020).

51. Zhao L, Lin Q, Wei J, Huai Y, Wang K, Yan HJIJoC*, et al.* CD44v6 expression in patients with stage II or stage III sporadic colorectal cancer is superior to CD44 expression for predicting progression. **8**, 692-701(2015).

52. Wang Z, von Au A, Schnolzer M, Hackert T, Zoller M. CD44v6-competent tumor exosomes promote motility, invasion and cancer-initiating cell marker expression in pancreatic and colorectal cancer cells. *Oncotarget* **7**, 55409-55436(2016).

53. Hartmans E, Orian-Rousseau V, Matzke-Ogi A, Karrenbeld A, de Groot DJ, de Jong S*, et al.* Functional Genomic mRNA Profiling of Colorectal Adenomas: Identification and in vivo Validation of CD44 and Splice Variant CD44v6 as Molecular Imaging Targets. *Theranostics* **7**, 482-492(2017).

54. Sakuma K, Sasaki E, Kimura K, Komori K, Shimizu Y, Yatabe Y*, et al.* HNRNPLL, a newly identified colorectal cancer metastasis suppressor, modulates alternative splicing of CD44 during epithelial-mesenchymal transition. *Gut* **67**, 1103-1111(2018).

55. Lv L, Liu HG, Dong SY, Yang F, Wang QX, Guo GL*, et al.* Upregulation of CD44v6 contributes to acquired chemoresistance via the modulation of autophagy in colon cancer SW480 cells. *Tumour Biol* **37**, 8811-8824(2016).

56. Mashita N, Yamada S, Nakayama G, Tanaka C, Iwata N, Kanda M*, et al.* Epithelial to mesenchymal transition might be induced via CD44 isoform switching in colorectal cancer. *J Surg Oncol* **110**, 745-751(2014).

57. Arabzadeh A, Dupaul-Chicoine J, Breton V, Haftchenary S, Yumeen S, Turbide C*, et al.* Carcinoembryonic Antigen Cell Adhesion Molecule 1 long isoform modulates malignancy of poorly differentiated colon cancer cells. *Gut* **65**, 821-829(2016).

58. Yamaguchi S, Yokoyama S, Ueno M, Hayami S, Mitani Y, Takeuchi A*, et al.* CEACAM1 is associated with recurrence after hepatectomy for colorectal liver metastasis. *J Surg Res* **220**, 353-362(2017).

59. Arabzadeh A, McGregor K, Breton V, Van Der Kraak L, Akavia UD, Greenwood CMT*, et al.* EphA2 signaling is impacted by carcinoembryonic antigen cell adhesion molecule 1-L expression in colorectal cancer liver metastasis in a cell context-dependent manner. *Oncotarget* **8**, 104330-104346(2017).

60. Dery KJ, Silver C, Yang L, Shively JE. Interferon regulatory factor 1 and a variant of heterogeneous nuclear ribonucleoprotein L coordinately silence the gene for adhesion protein CEACAM1. *J Biol Chem* **293**, 9277-9291(2018).

61. Huang X, Ye Q, Chen M, Li A, Mi W, Fang Y*, et al.* N-glycosylation-defective splice variants of neuropilin-1 promote metastasis by activating endosomal signals. *Nat Commun* **10**, 3708(2019).

62. Devaud C, Tilkin-Mariame AF, Vignolle-Vidoni A, Souleres P, Denadai-Souza A, Rolland C*, et al.* FAK alternative splice mRNA variants expression pattern in colorectal cancer. *Int J Cancer* **145**, 494-502(2019).

63. Lin JC, Lee YC, Tan TH, Liang YC, Chuang HC, Fann YC*, et al.* RBM4-SRSF3-MAP4K4 splicing cascade modulates the metastatic signature of colorectal cancer cell. *Biochim Biophys Acta Mol Cell Res* **1865**, 259-272(2018).

64. Rey C, Faustin B, Mahouche I, Ruggieri R, Brulard C, Ichas F*, et al.* The MAP3K ZAK, a novel modulator of ERK-dependent migration, is upregulated in colorectal cancer. *Oncogene* **35**, 3190-3200(2015).

65. Gadea G, Arsic N, Fernandes K, Diot A, Joruiz SM, Abdallah S*, et al.* TP53 drives invasion through expression of its Delta133p53beta variant. *Elife* **5**, (2016).

66. Zhao YJ, Han HZ, Liang Y, Shi CZ, Zhu QC, Yang J. Alternative splicing of VEGFA, APP and NUMB genes in colorectal cancer. *World J Gastroenterol* **21**, 6550-6560(2015).

67. Pentheroudakis G, Mavroeidis L, Papadopoulou K, Koliou GA, Bamia C, Chatzopoulos K*, et al.* Angiogenic and Antiangiogenic VEGFA Splice Variants in Colorectal Cancer: Prospective Retrospective Cohort Study in Patients Treated With Irinotecan-Based Chemotherapy and Bevacizumab. *Clin Colorectal Cancer* **18**, e370-e384(2019).

68. Rangwala F, Bendell JC, Kozloff MF, Arrowood CC, Dellinger A, Meadows J*, et al.* Phase I study of capecitabine, oxaliplatin, bevacizumab, and everolimus in advanced solid tumors. *Invest New Drugs* **32**, 700-709(2014).

69. Lin JC, Lee YC, Liang YC, Fann YC, Johnson KR, Lin YJ. The impact of the RBM4-initiated splicing cascade on modulating the carcinogenic signature of colorectal cancer cells. *Sci Rep* **7**, 44204(2017).

70. Kazemi M, Carrer A, Moimas S, Zandona L, Bussani R, Casagranda B*, et al.* VEGF121 and VEGF165 differentially promote vessel maturation and tumor growth in mice and humans. *Cancer Gene Ther* **23**, 125-132(2016).

71. Zaytseva YY, Elliott VA, Rychahou P, Mustain WC, Kim JT, Valentino J*, et al.* Cancer cell-associated fatty acid synthase activates endothelial cells and promotes angiogenesis in colorectal cancer. *Carcinogenesis* **35**, 1341-1351(2014).

72. Stagg BC, Uehara H, Lambert N, Rai R, Gupta I, Radmall B*, et al.* Morpholino-Mediated Isoform Modulation of Vascular Endothelial Growth Factor Receptor-2 (VEGFR2) Reduces Colon Cancer Xenograft Growth. *Cancers (Basel)* **6**, 2330-2342(2014).

73. Mizuno R, Chatterji P, Andres S, Hamilton K, Simon L, Foley SW*, et al.* Differential Regulation of LET-7 by LIN28B Isoform-Specific Functions. *Mol Cancer Res* **16**, 403-416(2018).

74. Makhafola TJ, Mbele M, Yacqub-Usman K, Hendren A, Haigh DB, Blackley Z*, et al.* Apoptosis in Cancer Cells Is Induced by Alternative Splicing of hnRNPA2/B1 Through Splicing of Bcl-x, a Mechanism that Can Be Stimulated by an Extract of the South African Medicinal Plant, Cotyledon orbiculata. *Front Oncol* **10**, 547392(2020).

75. Sillars-Hardebol AH, Carvalho B, Belien JA, de Wit M, Delis-van Diemen PM, Tijssen M*, et al.* BCL2L1 has a functional role in colorectal cancer and its protein expression is associated with chromosome 20q gain. *J Pathol* **226**, 442-450(2012).

76. Eilertsen IA, Sveen A, Stromme JM, Skotheim RI, Nesbakken A, Lothe RA. Alternative splicing expands the prognostic impact of KRAS in microsatellite stable primary colorectal cancer. *Int J Cancer* **144**, 841-847(2019).
